# Training non-surgeons to perform resuscitative thoracotomy

**DOI:** 10.1186/1757-7241-21-S1-A2

**Published:** 2013-05-28

**Authors:** T Konig, Z Perkins, GE Davies

**Affiliations:** 1 Defence Medical Services & London’s Air Ambulance, London UK; 2 Royal London Hospital & London’s Air Ambulance, UK

## 

Professor Moritz Schiff, a distinguished 19th century physiologist, provided the first description of thoracotomy, open cardiac massage and aortic compression for the resuscitation of cardiac arrest in 1874 [1] . In 1896, Ludwig Rehn performed the first successful thoracotomy, pericardotomy and cardiac wound repair on a young man dying from cardiac tamponade following a right ventricle stab wound [2]. Combined, these techniques make up the modern day resuscitative thoracotomy (RT), a classic damage control procedure.

RT is an immediate intervention for the injured patient in extremis or cardiac arrest with the cardinal objective of restoring adequate vital organ perfusion and controlling exsanguinating haemorrhage. The window for successful intervention is brief and as such it is frequently necessary to sacrifice optimal surgical conditions to achieve optimal results. To this end the procedure may need to begin during the pre-hospital phase or on Emergency Department arrival and be performed by non-cardiothoracic surgeons using basic equipment. However, if successful it is always completed in the operating theatre.

Without intervention, traumatic cardiac arrest carries a universally fatal prognosis. Resuscitative thoracotomy provides an opportunity for meaningful survival and observation of outcomes over the past 50 years has provided a clearer understanding of the patients that derive benefit and those in which intervention is futile. The key determinants of RT outcome are the mechanism and site of injury and the duration of cardiac arrest. Approximately 10% of patients with penetrating injuries survive compared to less than 2% of those with a blunt mechanism of injury.^3 ^^4^ Importantly, survival is significantly higher in those with stab wounds (17%-24%) than those with gun shot wounds (3%-4%) [3,5]. The best outcomes are seen in patients with penetrating cardiac wounds with up to one third surviving [3,4,6].

Despite the severe pathophysiological insult, complete neurological recovery is common in survivors, with only a minority (8% - 15%) suffering neurological impairment [3,4,7]. Survival is directly correlated with the duration of cardiac arrest. RT is futile if CPR has been required for greater than 15 minutes following penetrating trauma or 10 minutes following blunt trauma, without any response [8,9]. Within these limits the cardiac rhythm is not a discriminator of futility, with multiple survivors from asystole described [7-9]. Flexibility needs to exist to permit the provision of care to come forward to meet the patient. If the emergency department has sufficient notification by Emergency Medical Service pre-alert then provision can be made to move the patient to an operating room as quickly as possible. If, however, the patients physiological state cannot tolerate further delay then the procedure may need to be carried out in the resuscitation room. On occasion the pre hospital time exceeds these time limits and so RT may need to be carried out at the point of wounding. Physician staffed pre hospital care teams like those that make up a large number of the United Kingdom Helicopter Emergency Medical Service (HEMS) teams have been called on to perform RT before the patient is transferred to hospital on an ever increasing number of occasions. The success rates when this occurs are in the region of 18% [7]. A survival rate that compares favourably with outcomes from in-hospital intervention.

To this end, a number of physicians from a variety of non-surgical specialties need to be trained to perform RT. Decision to intervene, wielding a scalpel and attempting to fix what has been damaged can be an intimidating venture for the infrequent surgeon. Consideration should be made to personal safety, futility, training, understanding of the procedure, governance, colleague criticism, professional ability and patient dignity and care. All these things may need to be considered in the short period of time before the patient succumbs to their injuries.

To meet this need, a number of courses have been developed to give insight, provide academic and intellectual support and most importantly provide hands on training to these providers in advance of the occasion when they first come face to face with a patient who needs immediate resuscitative thoracotomy. As a result these patients are more promptly and better served and outcomes improved.One such course is the Pre Hospital Emergency Resuscitative Thoracotomy (PERT) course run at the Royal College of Surgeons of England (http://www.rcseng.ac.uk/courses/course-search/resuscitative-thoracotomy). This course provides expert teaching on the subject from a varied faculty of Emergency Physicians, Anaesthetists and Surgeons, with a highly credible combined RT experience. The day long course focuses on the disease itself and the public health issues surrounding penetrating trauma, the anatomy of the thorax, decision making, pitfalls and service implementation before an afternoon when the students are shown methods of opening the chest, relieving cardiac tamponade, methods of myocardial repair and thoracic haemorrhage control. At all times the teaching is centred on case based discussion to give perspective to the procedure.

Another introduction to resuscitative thoracotomy occurred at this years London Trauma Conference. A break out workshop on the many aspects of RT was again provided by an expert faculty to a number of non-surgeons from an international audience. A background to the procedure was supported by an insight into the anatomy, advice about optimal team preparation and a simulated demonstration of a pre hospital case ‘moulage’ on a manikin. Students were shown and allowed to practice myocardial suture repair, discuss cases and gain insight into the post operative care of patients after successful thoracotomy.

These insights and courses are aimed at empowering clinicians to identify and apply a beneficial intervention to those that need it, when they need it. Ludwig Rehn’s pertinent conclusion following the first successful RT over a century ago remains: “many lives can be saved that were previously counted as lost [2].”

## References

[B1] Vallejo-Manzur F Varon J Fromm R Jr. Baskett P Moritz Schiff and the history of open-chest cardiac massage Resuscitation 2002 53 1 3 5 11947972 10.1016/s0300-9572(02)00028-x

[B2] Blatchford JW 3rd Ludwig Rehn the first successful cardiorrhaphy Ann Thorac Surg 1985 39 5 492 5 3888132 10.1016/s0003-4975(10)61972-8

[B3] Rhee PM Acosta J Bridgeman A Wang D Jordan M Rich N Survival after emergency department thoracotomy: review of published data from the past 25 years J Am Coll Surg 2000 190 3 288 98 10703853 10.1016/s1072-7515(99)00233-1

[B4] Practice management guidelines for emergency department thoracotomy. Working Group, Ad Hoc Subcommittee on Outcomes, American College of Surgeons-Committee on Trauma J Am Coll Surg 2001 193 3 303 9 11548801 10.1016/s1072-7515(01)00999-1

[B5] Seamon MJ Shiroff AM Franco M Stawicki SP Molina EJ Gaughan JP Emergency department thoracotomy for penetrating injuries of the heart and great vessels: an appraisal of 283 consecutive cases from two urban trauma centers J Trauma 2009 67 6 1250 7 discussion 57-8 20009674 10.1097/TA.0b013e3181c3fef9

[B6] Burlew CC Moore EE Moore FA Coimbra R McIntyre RC Jr. Davis JW Western Trauma Association Critical Decisions in Trauma: Resuscitative thoracotomy J Trauma Acute Care Surg 2012 73 6 1357 61 10.1097/TA.0b013e318270d2df 23188227

[B7] Davies GE Lockey DJ Thirteen survivors of prehospital thoracotomy for penetrating trauma: a prehospital physician-performed resuscitation procedure that can yield good results J Trauma 2011 70 5 E75 8 21131854 10.1097/TA.0b013e3181f6f72f

[B8] Moore EE Knudson MM Burlew CC Inaba K Dicker RA Biffl WL Defining the limits of resuscitative emergency department thoracotomy: a contemporary Western Trauma Association perspective J Trauma 2011 70 2 334 9 21307731 10.1097/TA.0b013e3182077c35

[B9] Powell DW Moore EE Cothren CC Ciesla DJ Burch JM Moore JB Is emergency department resuscitative thoracotomy futile care for the critically injured patient requiring prehospital cardiopulmonary resuscitation? J Am Coll Surg 2004 199 2 211 5 15275875 10.1016/j.jamcollsurg.2004.04.004

